# Skin microbiome differences in pancreatic adenocarcinoma, other cancers, and healthy controls: a pilot study

**DOI:** 10.3389/fonc.2025.1495500

**Published:** 2025-02-06

**Authors:** Taylor Davis, Katherine T. Decker, Dana Hosseini, Gayle Jameson, Erkut Borazanci

**Affiliations:** ^1^ Department of Oncology, HonorHealth Research Institute, Scottsdale, AZ, United States; ^2^ ProdermIQ, Inc., San Diego, CA, United States

**Keywords:** skin microbiome, pancreatic adenocarcinoma, dysbiosis, machine learning, alpha diversity

## Abstract

**Introduction:**

Many studies have reported the importance of the human microbiome in relationship to the overall health of its host. While recent studies have explored the microbiome’s role in various types of cancer compared to healthy patients, this pilot study is the first to investigate differences in the skin microbiome composition among pancreatic adenocarcinoma patients, individuals with other cancers, and cancer-free controls.

**Methods:**

The study characterizes the skin microbiome’s potential associations with cancer status by analyzing skin swabs from the forehead and cheek of 58 participants using Next Generation Sequencing (NGS), differential abundance analysis, and machine learning techniques.

**Results:**

The study results indicated that the cancer group displayed a significantly higher mean alpha diversity compared to the control group. Additionally, a machine learning classification model achieved a mean F1 Score of 0.943 in predicting cancer status, indicating measurable differentiation in the skin microbiome between the study groups. This differentiation is supported by differential abundance methods, including ANCOM-BC and MaAsLin2.

**Discussion:**

This pilot study suggests that skin microbiome profiling could serve as a non-invasive biomarker for cancer detection and monitoring, which warrants a larger, longitudinal study to validate these results.

## 1 Introduction

The human skin, the body’s largest organ, functions as the protective barrier against external elements and hosts a diverse community of microorganisms collectively known as the skin microbiota. These microorganisms, which inhabit sebaceous, dry, and moist microenvironments, are primarily harmless or even beneficial, although some can pose a threat (1, 2). The collective genetic material, or genome, of these microbes is defined as the skin microbiome (3). Differences in the microbiome can be influenced by many factors such as age, occupation, sex, and geographical location (4). The microbiome has many functions, such as developing the immune system, digesting food, and maintaining a protective barrier. Disruptions to the microbiome, or dysbiosis, have been implicated in various diseases, reinforcing the importance of understanding its characteristics and functions in health and disease (5).

Previous research has demonstrated that the human microbiome plays a significant role in the overall fitness of the host. The microbiome has been linked to a broadening array of health issues, including, but not limited to: inflammatory bowel disease (IBD), Crohn’s disease, eczema, psoriasis, autism, depression, anxiety, obesity, acne, diabetes, hypertension, chronic sinusitis, dental caries, and bacterial vaginosis (5). In addition to these health issues, recent studies have explored the microbiome’s role in various types of cancer compared to healthy patients. For example, patients with conventional adenoma and colorectal cancer have shown to have less diversity in gut microbiota than that of healthy patients, whereas those with sessile serrated adenoma had more diversity in gut microbiota than that of healthy patients (6). Evidence also suggests that the gut and skin microbiome are critical in regulating the ability of immune checkpoint inhibitors in the treatment of skin cancers (7). Additionally, some studies have reported significant differences in microbial diversity between patients with breast cancer and healthy individuals (8). Building on these insights, this study narrows its focus to pancreatic cancer. Alterations in microbiome diversity, proportions, and dominant species have been associated with pancreatic cancer development (9), and indirect evidence suggests a potential link specifically between the skin microbiome and pancreatic cancer (10), highlighting the importance of further investigation into its specific microbial dynamics.

The purpose of this study was to characterize the skin microbiomes on the forehead and cheek of individuals with pancreatic adenocarcinoma compared to those with other forms of cancer and individuals without cancer. The skin profiling platform developed by ProdermIQ, Inc. was used to explore the differences in bacterial diversity and composition of the skin microbiome between the study groups. The goal of this pilot study was to determine if results from this trial could provide insight on the associations between microbial flora and cancer status. Future studies will aim to explore the links between the microbiome and cancer severity, status of host immune system, progress of an ongoing therapy, and implications for therapeutic applications.

## 2 Methods

### 2.1 Study subjects and clinical examination

The study received Institutional Review Board (IRB), and all participants provided informed consent prior to enrollment. Patients, staff, and family members within the Debi and Jerry Research Bisgrove Research Pavilion at HonorHealth Research Institute (Scottsdale, Arizona) were invited to participate. Following consent, individuals were assigned an identification number to maintain deidentification, which was linked to their skin swab sample. Participants also completed a questionnaire that collected their age, gender, ethnicity, race, weight, and height, as well as details regarding current skin conditions, medications, and skincare product use.

To supplement the study, an additional 60 healthy control samples were drawn from an existing broader database of ProdermIQ’s healthy skin samples that were generated with the same experimental protocol as the samples in this study. These control samples were carefully chosen to minimize confounding variables, ensuring that the age and gender distribution of these additional samples closely matched that of the cancer group.

A total of 58 study participants were divided into three groups: cancer patients with pancreatic adenocarcinoma (n=23), cancer patients with other types of cancer (n=21), and individuals without cancer (n=14). The other forms of cancer included breast cancer (n=13), head and neck cancer (n=1), rectal cancer (n=1), colon cancer (n=1), peritoneal cancer (n=1), sarcoma (n=1), lung cancer (n=1), endometrial cancer (n=1), and unspecified cancer (n=1).

Enrolled individuals met the following inclusion criteria: (i) adults at least 18 years of age; (ii) the ability to understand the requirements of the study, and provide written informed consent; (iii) for subjects defined as pancreatic adenocarcinoma must have a histological confirmed diagnosis of pancreatic adenocarcinoma; (iv) for subjects defined as other cancer, must have a histological confirmed diagnosis of a malignancy other than pancreatic adenocarcinoma; (v) for subjects defined as no malignancy, these individuals must no history of any type of cancer. Exclusion criteria included: (i) patients with a rash or skin disorder of any kind on their face; (ii) allergy to cotton swabs.

### 2.2 DNA preparation and sequencing

ProdermIQ provided sample collection kits to HonorHealth. Each kit contained one vial of sterile water (indicated with a “W” on the lid), two vials of fixative solution (one labeled “FH” for the forehead and one labeled “CK” for the cheek), and sterile cotton swabs. Prior to the day of collection, participants were instructed not to wash their face, wear any makeup, or use any facial cleansers and lotions at least 8 hours prior to collection. Using the provided swabs, patients were instructed to swab their forehead and cheek in a circular fashion about the size of a quarter prior to placing each swab into the corresponding fixative solution. Once collected, the swab samples were stored in a freezer with a temperature between 4°F-32°F. The samples were then shipped to ProdermIQ for Next Generation Sequencing (NGS) amplicon sequencing to characterize the skin microbiomes.

The methods of sequencing are described in detail elsewhere (11). Briefly, samples were processed using a proprietary skin microbiome profiling panel (ProdermIQ) that targeted multiple regions of the 16S rRNA gene, including the V1, V2, V4, V6, V7, V8, and V9 regions. Sequencing was performed on the Illumina MiSeq platform using 300 base pair paired-end reads. ProdermIQ’s custom quality control and read processing steps were used to preprocess the raw sequencing data and identify amplicon sequence variants (ASVs). Taxonomic classification was performed using ProdermIQ’s reference database, which is based on the Integrated Microbial Genomes (IMG) database (12).

### 2.3 Data analysis

The analysis of skin microbiome samples incorporated diversity metrics, statistical methods, and machine learning (ML) techniques.

#### 2.3.1 Microbiome diversity

##### 2.3.1.1 Alpha diversity

Alpha diversity, representing the microbial diversity within individual samples, was assessed through three measures:

1. Observed Features: The total number of distinct taxa.

2. Shannon’s Diversity Index: A measure including both richness and evenness, which is calculated as:


$$H\;=\;-\sum_{i=1}^{S}p_{i}\log_{2}p_{i}$$

where *S* is the total number of taxa, and *p_i_* is the proportion of each taxa *i* in the sample.

3. Simpson’s Diversity Index: A measure which similarly includes richness and evenness, and emphasizes evenness by evaluating the likelihood that two randomly chosen individuals originate from different taxa:


$$D\;=\;1\;-\;\sum_{i=1}^{S}{p_{i}}^{2}$$

To compare alpha diversity metrics between groups (cancer versus control), the Mann-Whitney U test was applied.

##### 2.3.1.2 Beta diversity

Beta diversity, quantifying the differences in microbial community composition between samples, was assessed using two measures:

1. Jaccard Distance: The proportion of taxa that do not have the same binary presence/absence value between two samples.

2. Bray-Curtis Dissimilarity: A measure that assesses the microbial abundance differences across two samples, *u* and *v*:


$$d\left(u,v\right)\;=\;\frac{\sum_{i}\left|u_{i}+v_{i}\right|}{\sum_{i}\left|u_{i}+v_{i}\right|}$$

The resulting distance matrices were visualized using Principal Coordinate Analysis (PCoA), and the differences between the control and cancer samples were statistically evaluated using Permutational Multivariate Analysis of Variance (PERMANOVA). Pseudo-F statistics and p-values were reported to assess group differences.

The diversity metrics were computed using the “skbio.diversity” module in Python.

#### 2.3.2 Statistical analysis of subject characteristics

Statistical comparisons of subject characteristics, including age and the number of medications, were conducted to investigate differences between pancreatic cancer and other cancer groups. The Mann-Whitney U test was used for both analyses. Specifically, the age distributions and number of medications taken by subjects were compared between the two groups under the null hypothesis that their distributions are identical.

#### 2.3.3 Multiple testing correction

Given the multiple comparisons made across the different metrics, the Bonferroni correction was applied to adjust the significance levels. Corrected p-values were calculated using the *multipletests* function from the “statsmodels.stats.multitest” module in Python.

#### 2.3.4 Machine learning using taxonomic count data

To differentiate between cancer (all types) and healthy control statuses, machine learning classification models were developed using a Support Vector Machine (SVM) framework. The models were trained on the relative abundance of taxa in the skin microbiome to identify key microbial features associated with the cancer and control cohorts. Prior to analysis, the relative abundance data was cleaned to include only taxa present in at least five samples, a threshold chosen to balance the exclusion of rare taxa that may represent noise or sequencing artifacts with the retention of taxa likely to provide meaningful biological insights. Potential contaminants were identified and removed based on a reference database of expected skin microbes. However, taxa with relative abundances exceeding 30% were retained, regardless of their presence in the skin reference database, to ensure biologically relevant signals were not excluded.

Feature selection was performed using Recursive Feature Elimination with Cross-Validation (RFECV) to reduce dimensionality and improve model performance. RFECV iteratively removed less informative features while retaining those that provided the most predictive power. Comparing 150 random seeds of RFECV feature selection led to the identification of a robust set of 41 taxa.

To evaluate the models’ effectiveness, 10-fold cross-validation was repeated over 1,000 iterations of random train-test splits. In each iteration, group-stratified sampling ensured that both cancer and control groups were proportionally represented in the training and test sets. Model performance metrics were calculated using the “sklearn” module in Python, and the mean and standard deviation of the metrics across the iterations were calculated.

#### 2.3.5 Differential abundance

##### 2.3.5.1 ANCOM-BC

Differential abundance analysis was performed using the ANCOM-BC (Analysis of Compositions of Microbiomes with Bias Correction) (13) method through the QIIME2 (14) plugin. ANCOM-BC was applied to the unnormalized, cleaned taxa counts to identify taxa with significant differences in abundance between the cancer and control groups. A q-value threshold of 0.05 was used to determine statistical significance.

##### 2.3.5.2 MaAsLin2

Differential abundance analysis was also performed using MaAsLin2 (Microbial Multivariable Association with Linear Models) (15) to identify taxa significantly associated with cancer and control groups. MaAsLin2 was run with default parameters, except for the q-value threshold, which was adjusted to 0.05 for statistical significance. The model included fixed effects for class (study group: cancer or control), sample origin (this study or broader ProdermIQ healthy dataset), age, and gender. To account for repeated measures and variability in sampling location, random effects were applied for subject and swab site.

## 3 Results

### 3.1 Study participants and cohort characteristics

A total of 58 participants were enrolled in this study to characterize the skin microbiomes across three cohorts: pancreatic adenocarcinoma, other cancer, and no cancer (controls). Characteristics of the subjects within each cohort, including average age, gender, smoking status, and ethnicity, are summarized in **Table 1**. Among the no cancer cohort, 4 participants did not indicate their age, gender, smoking status, or ethnicity. A total of 150 samples were analyzed, including 79 samples from subjects with cancer and 71 from control subjects. Of the 71 control samples, 60 were sourced from a broader database of ProdermIQ healthy skin samples that were generated using the same experimental protocol as this study. These 60 additional samples were selected to minimize bias in comparing healthy and cancer-affected skin microbiomes by focusing on subjects in good health, matching swab sites from this study (cheek and forehead), and balancing age and gender distributions to mirror those of the cancer group.

**Table 1:** Demographic and clinical characteristics of subjects.

	Pancreatic Adenocarcinoma (n=23)	Other Cancer (n=21)	No Cancer, HonorHealth subjects (n=14)	No Cancer, ProdermIQ (n=41)
*Characteristics of subjects*				
Average Age (yr.)	65.8	59.8	39.6	58.0
Gender (M/F/Unlisted)	12/11/0	5/16/0	3/7/4	18/23/0
Smoker (Y/N/Unlisted)	1/22/0	0/21/0	0/10/4	0/22/19
Ethnicity (Caucasian/Hispanic or Latino/Asian/African American/Native American/Unlisted	20/1/0/1/0/0	13/5/3/0/0/0	8/0/0/1/1/4	39/0/1/0/0/1

**Figure 1:**
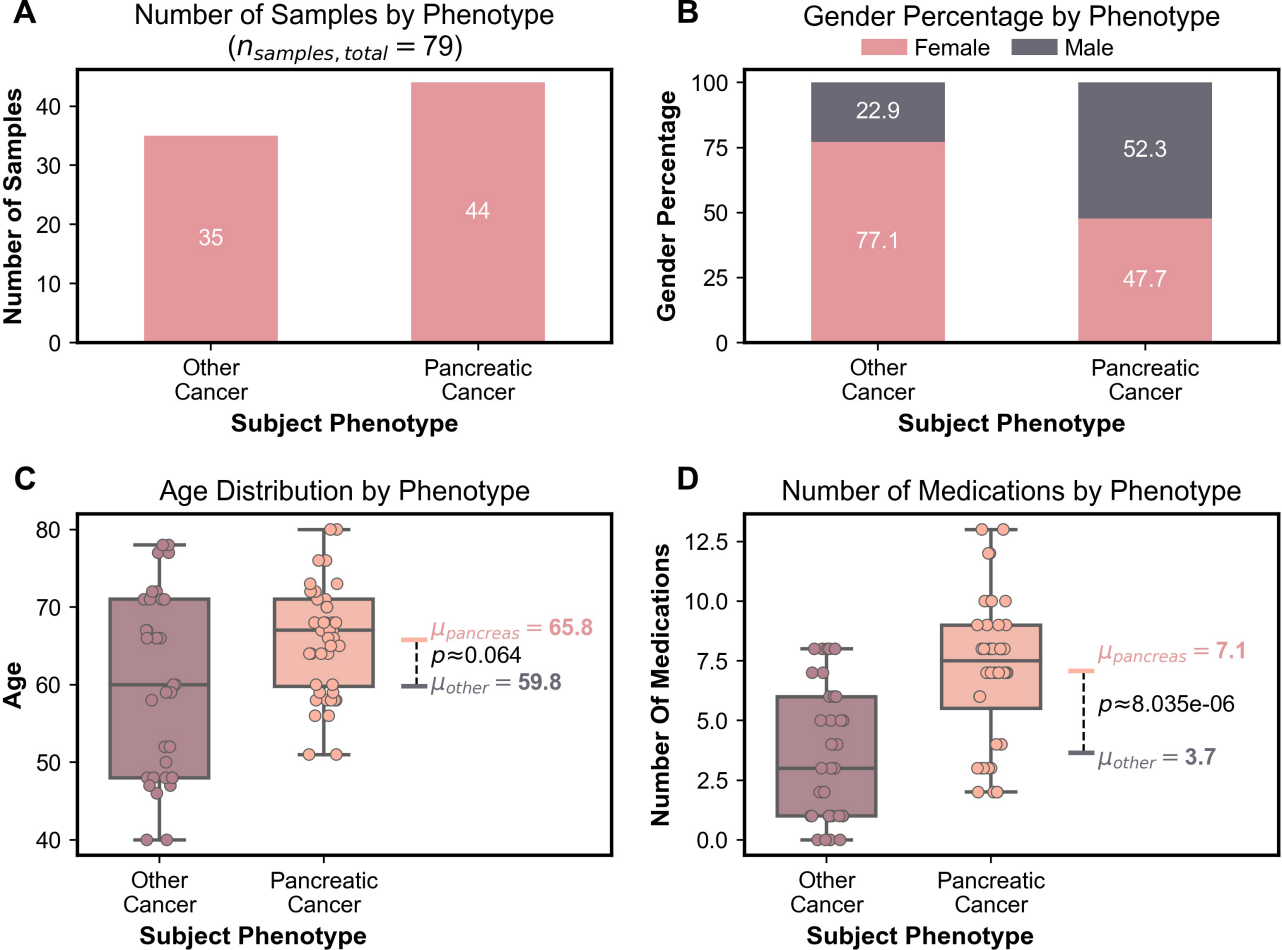
**(A)** Bar graph depicting number of samples in each cancer group. **(B)** Stacked bar graph representing gender percentage for each cancer group. **(C)** Boxplots representing age distribution including the mean age and the Bonferroni corrected Mann-Whitney U test p-value for the two cancer groups. **(D)** Boxplots representing the number of medications including the mean number of medications and the Bonferroni corrected Mann-Whitney U test p-value for the two cancer groups.

Within the cancer group, the pancreatic adenocarcinoma cohort contained 44 samples and the other cancer cohort contained 35 samples. There were significant differences between the two cancer groups in terms of gender distribution, mean age, and medication count. The pancreatic adenocarcinoma cohort was predominantly male with 52.3% male subjects and 47.7% female subjects. In contrast, the other cancer group was predominantly female with 77.1% female subjects and 22.9% male subjects. The mean age of the pancreatic adenocarcinoma group trended higher with a mean age 65.8 years and 59.8 years for the other cancer group. The difference in the number of medications between the groups also showed significance. At the time of sampling, the pancreatic adenocarcinoma group was taking an average of 7.1 medications while the other cancer group was taking an average of 3.7 medications (p=1.57x10^-6^), as shown in **Figure 1**.

### 3.2 Diversity measures

A total of 11,268 amplicon sequence variants (ASVs) were identified following ProdermIQ’s custom preprocessing pipeline. Of these, 11,109 ASVs (98.6%) were successfully mapped to a microbial taxon at the domain level, including 10,839 Bacteria and 270 Archaea. At the subspecies level, 9,931 ASVs (88.1%) were assigned, representing nearly all of the sequencing reads (99.33%). These subspecies-level assignments corresponded to 2,736 unique taxa, which were used as the basis for following diversity analyses and other investigations. However, it is important to note that the subspecies-level assignments represent the closest known strains based on the available reference database and may not correspond to the exact strains present *in situ*.

**Figure 2:**
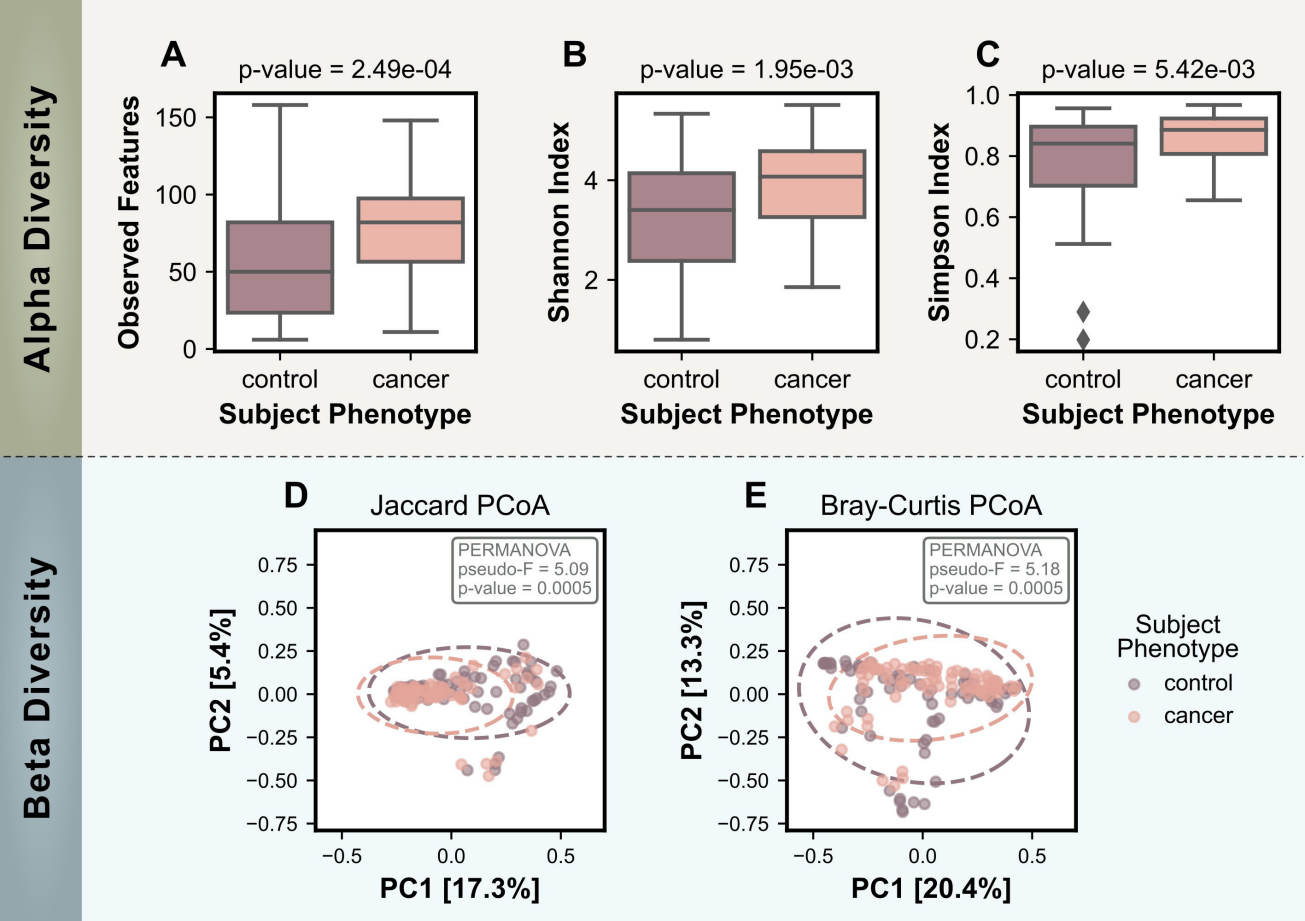
Comparison of alpha and beta diversity measures for control (no cancer) and cancer groups. Alpha diversity measures include: **(A)** the number of observed features, **(B)** the Shannon Index, and **(C)** the Simpson Index. Bonferroni-corrected p-values for statistical differences between the two groups were calculated using the Mann-Whitney U test and are displayed on the plots. Beta Diversity PCoA plots are based on **(D)** the Jaccard Distance and **(E)** the Bray-Curtis Dissimilarity, with ovals representing a 95% confidence interval. Statistical significance for beta diversity differences was assessed using PERMANOVA and p-values are shown on the plots.

Microbial diversity within and between samples was assessed using alpha and beta diversity metrics. Alpha diversity metrics, which evaluate the richness and evenness of microbial taxa within individual samples, were significantly higher in the cancer group compared to the control group (**Figures 2A–C**). This trend was consistent across all three metrics used: observed features (p-value =2.49x10^-4^), Shannon Diversity Index (p-value=1.95x10^-3^), and Simpson Diversity Index (p-value=5.42x10^-3^). These results indicate a greater variety of microbes on the skin of cancer subjects.

Beta diversity analyses, which evaluate differences in microbial community composition between samples, further supported the distinction between the cancer and control skin microbiomes. Both a presence/absence based method (Jaccard distance) and an abundance-based method (Bray-Curtis dissimilarity) were used to calculate dissimilarity matrices that were visualized using PCoA in **Figures 2D, E**. PERMANOVA tests reported statistically significant differences between the cancer and control groups for both metrics (Jaccard: pseudo-F = 5.09, p-value = 0.0005; Bray-Curtis: pseudo-F = 5.18, p-value = 0.0005).

The beta diversity PCoA plots showed that the control group included a broader spread of data points, reflected by the larger 95% confidence interval ovals. In contrast, the cancer group displayed tighter clustering, which suggests less variability among cancer-associated skin microbiomes. Notably, the cancer group oval appeared to be a subset of the larger control group oval for both metrics, indicating the microbial communities associated with cancer could represent a subset of the broader beta diversity in controls.

In an additional analysis, the diversity metrics and statistical analyses were conducted on various other subject metadata fields, including gender, age, and number of medications. For all three of these subject metadata fields, significant differences in alpha diversity were not consistently detected across the various alpha diversity metrics. However, female samples were found to be more diverse using the Shannon and Simpson Indices, and age showed positive Spearman correlation with the observed numbers of features. In contrast, all three subject metadata fields were found to have consistently significant differences in beta diversity using PERMANOVA. This result suggests that these subject characteristics do influence the overall composition of the microbiome.

### 3.3 Top abundance organisms

**Figure 3:**
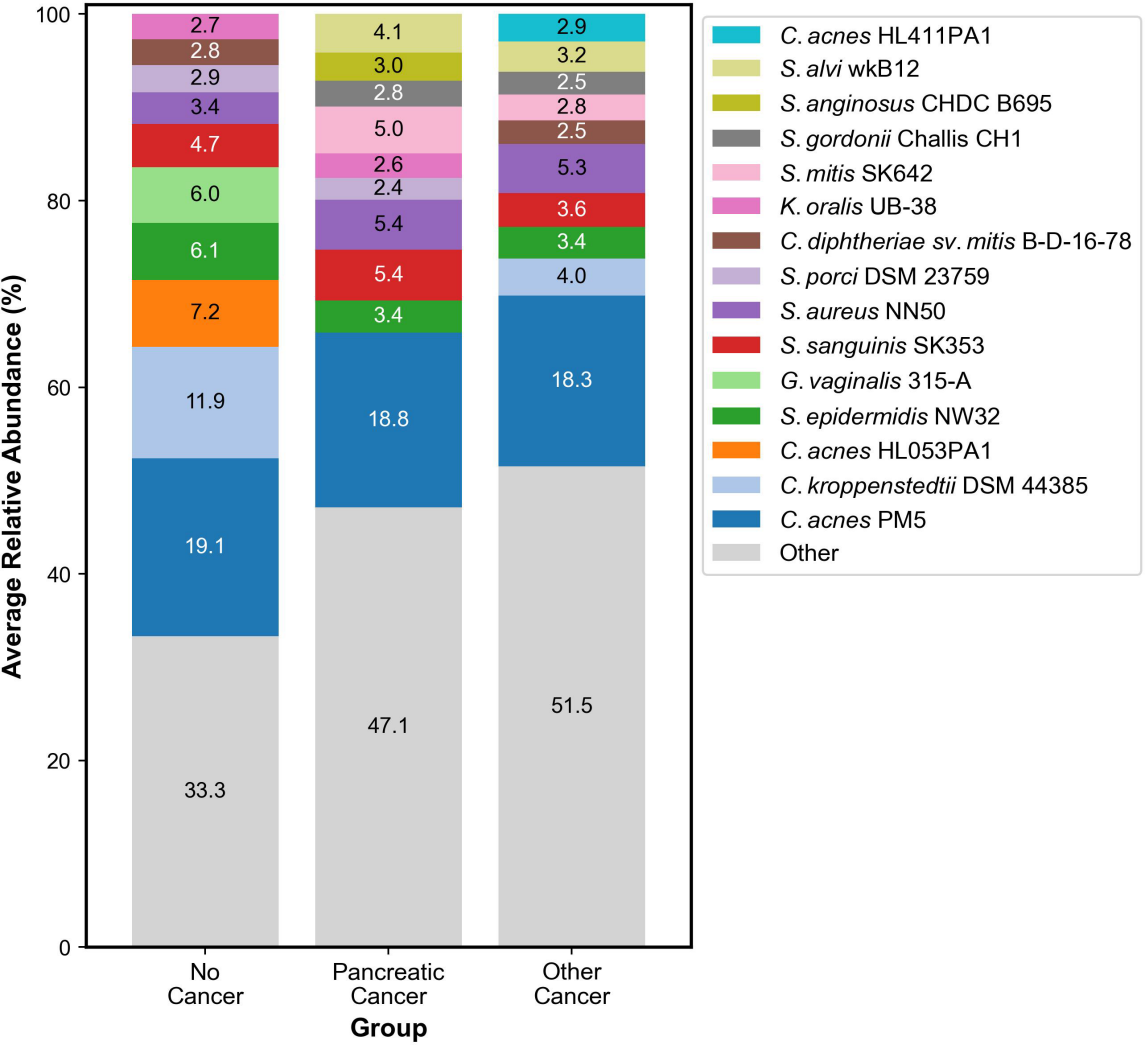
Stacked bar graph representing the ten most abundant organisms in the skin microbiome within each group.

Our analysis showed that the following organisms were the most abundant across all samples: *Cutibacterium acnes* PMH5, *Streptococcus sanguinis* SK353, *Staphylococcus aureus* subsp. *aureus* NN50, *Streptococcus mitis SK*642, *Snograssella alvi* wkB12, *Staphylococcus epidermidis NW*32, *Streptococcus anginosus* ChDC B695, *Streptococcus gordonii* Challis CH1, *Kingella oralis* UB-38, *Streptococcus porci* DSM 23759, *Cutibacterium acnes* HL411PA1, *Corynebacterium kroppenstedtii* DSM 44385, *Corynebacterium diphtheriae* sv. *mitis* B-D-16-78, *Gardnerella vaginalis* 315-A, and *Cutibacterium acnes* HL053PA1, as shown in **Figure 3**. Organisms such as *Gardnerella vaginalis* 315-A and *Cutibacterium acnes* HL053PA1 were seen in abundance within the no cancer group. Other organisms such as *Streptococcus mitis* SK642, *Snograssella alvi* wkB12, and *Streptococcus gordonii* Challis CH1 were seen in abundance within the pancreatic adenocarcinoma and other cancer groups but not within the no cancer group. *Streptococcus porci* DSM 23759 and *Kingella oralis* UB-38 were seen significantly within the pancreatic adenocarcinoma and no cancer groups but not within the other cancer group. *Corynebacterium kroppenstedtii* DSM 44385 and *Corynebacterium diphtheriae* sv. *mitis* B-D-16-78 were seen significantly in the other cancer and no cancer groups but not within the pancreatic adenocarcinoma group. *Streptococcus anginosus* ChDC B695 was seen in abundance only within the pancreatic adenocarcinoma group. *Cutibacterium acnes* HL411PA1 was seen in abundance only within the other cancer group.

### 3.4 Machine learning classifier

**Figure 4:**
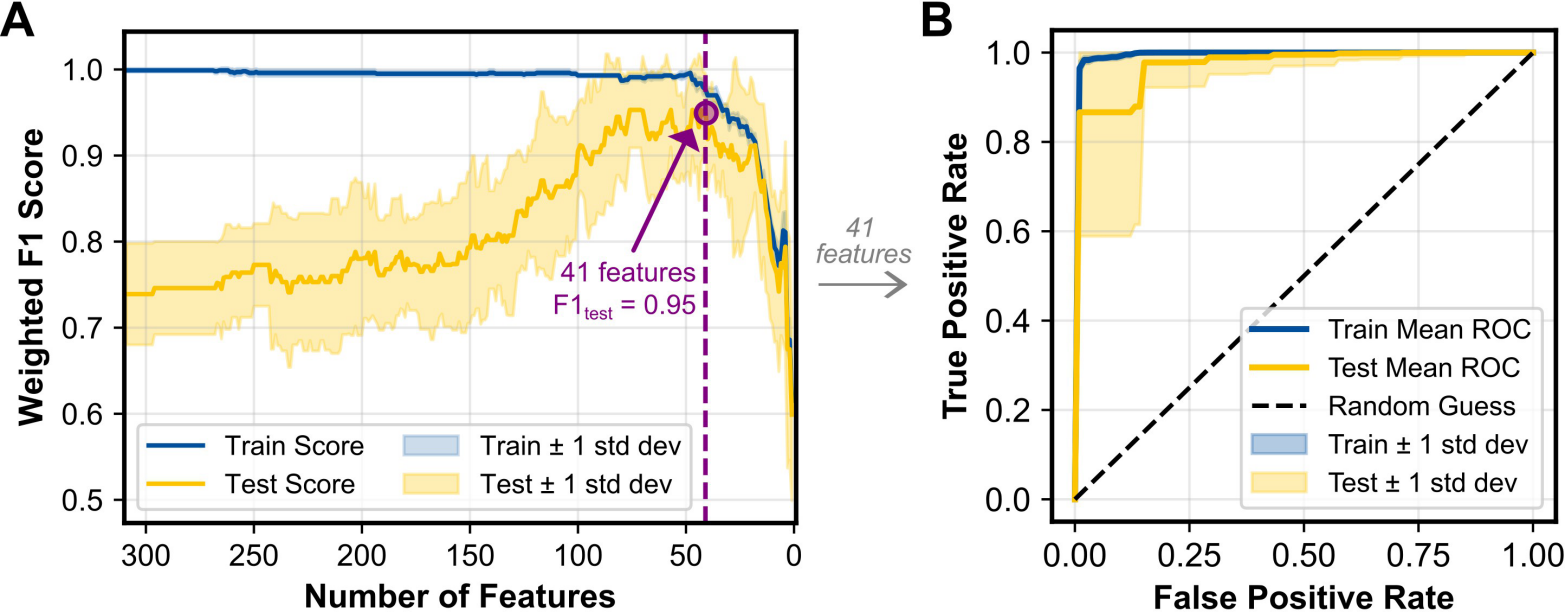
**(A)** RFECV results showing the weighted F1 score for the training and test datasets as a function of the number of features included in the model. The purple dashed line indicates the number of features (41 taxa) that achieved the maximum F1 score and was selected for additional analysis. **(B)** ROC curves for the training and test datasets, averaged across 1,000 iterations. The diagonal dashed line represents random classification performance (AUC = 0.5). Shaded regions represent ±1 standard deviation for both panels.

To assess the predictive capability of the skin microbiome for cancer detection, a machine learning binary classification model was developed, utilizing the relative abundances of the skin microbiome bacterial composition. Pancreatic adenocarcinoma and other cancer types were combined into a single “cancer” group to ensure sufficient data for training the model, while the no cancer samples were categorized as the “control” group. The model achieved a mean F1 score of 0.943 in a 10-fold cross validation process across 1,000 randomly generated training and testing splits. For each of these splits, group-stratified splitting ensured an approximately equal number of cancer and control samples in each test set. Classification performance was similarly strong across other metrics, including accuracy, sensitivity, specificity, error rate, and receiver operating characteristic (ROC) area under the curve (AUC) (**Table 2**). The classification model’s strong proficiency was enabled by recursive feature elimination (RFE), which identified an optimal set of 41 taxa to minimize noise while preserving key signals (**Figure 4A**). The average ROC curves from 1,000 iterations with the selected 41 taxa indicates strong performance for both training and test data (**Figure 4B**).

**Table 2:** Machine learning classification performance using 10-fold cross validation across 1,000 random seeds of train/test data splitting.

	Training Data (Mean ± Std Dev)	Testing Data (Mean ± Std Dev)
*Metric*		
Accuracy	0.978 ± 0.008	0.943 ± 0.059
Sensitivity	0.966 ± 0.012	0.930 ± 0.090
Specificity	0.992 ± 0.008	0.959 ± 0.075
Error Rate	0.022 ± 0.008	0.057 ± 0.059
ROC AUC	0.998 ± 0.001	0.976 ± 0.046
Weighted F1 Score	0.978 ± 0.008	0.943 ± 0.060

### 3.5 Key members of skin microbiome associated with cancer and control

**Figure 5:**
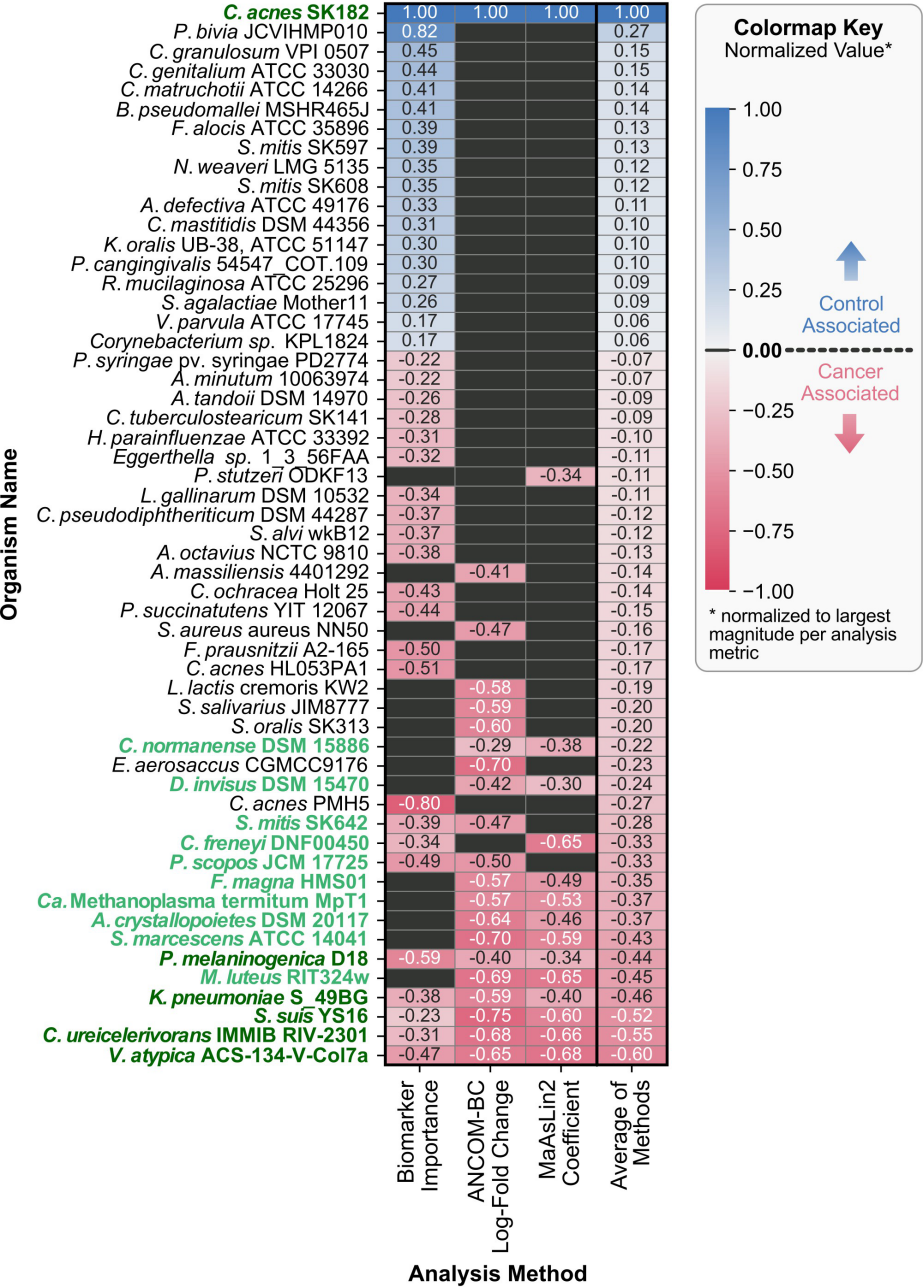
Organism directionality and normalized metrics across various methods. Each row represents a unique organism identified across the three analyses, with columns corresponding to the methods used: ML-derived biomarker selection, ANCOM-BC, and MaAsLin2. The color of each cell indicates the direction of association (red for cancer-associated, gray for no significant association, and blue for control-associated), with darker pigments corresponding to larger normalized values of biomarker importance, LFC for ANCOM-BC, effect size (coefficient) for MaAsLin2. The arithmetic mean of the normalized values is displayed in the rightmost column (“Average of Methods”), which was also used to sort the rows of the heatmap. For organisms not identified as significantly associated by a method, their values were set to zero before averaging. Y-axis labels are formatted to highlight taxa appearing in multiple analyses (bold and dark green for all three, bold and lighter green for two out of three).

The machine learning classification model described in Section 3.4 identified and ranked the key microbial features, or “biomarkers,” distinguishing the cancer group from the control group. To further investigate the key microbes, the machine learning results have been integrated with differential abundance approaches, including ANCOM-BC and MaAsLin2, and the superset of significant results across the three methods are visualized in **Figure 5**.

#### 3.5.1 Machine learning-derived biomarkers

Recursive feature elimination with cross-validation (RFECV) identified a set of 41 taxa that provided sufficient predictive information while minimizing noise. Across the 1,000 iterations of train-test splits used to rank the 41 taxa, *Cutibacterium acnes* SK182, associated with the control group, consistently ranked at or near the top for feature importance. In contrast, the highest-ranking cancer-associated taxon was a distinct strain of the same species, *Cutibacterium acnes* PMH5. The feature importances for the 41 selected taxa are available in **Supplementary Table S1**.

#### 3.5.2 Differential abundance analysis with ANCOM-BC

ANCOM-BC identified 21 taxa with significant differences in abundance between the cancer and control groups. Taxa with positive log-fold changes (LFC) were enriched in the control group, while taxa with negative LFC values were enriched in cancer samples. Consistent with the machine learning findings, *Cutibacterium acnes* SK182 showed the largest enrichment in the control group (LFC = 2.01, q-value = 3.36x10^-5^). Notably, all remaining significantly differential taxa were associated with the cancer group, with *Streptococcus suis* YS16 showing the largest negative LFC (LFC = -1.50, q-value = 2.63x10^-6^). All significant results from ANCOM-BC are available in **Supplementary Table S2**.

#### 3.5.3 Differential abundance analysis using MaAsLin2

MaAsLin2 analysis identified 15 taxa as significantly associated with the cancer and control groups while accounting for covariates including age and gender. The directionality of the associations was determined by the sign of the coefficients, where positive values indicated enrichment in the control group and negative values indicated enrichment in the cancer group. *Cutibacterium acnes* SK182 emerged as the top control-associated taxon (coefficient = 3.51, q-value = 1.72x10^-4^), which aligns with findings from both the machine learning analysis and ANCOM-BC. The MaAsLin2 results also mirrored the trend observed in ANCOM-BC, with most significant taxa being cancer-associated, except for *Cutibacterium acnes* SK182. For the cancer group, *Veillonella atypica* ACS-134-V-Col7a was the enriched taxon with the largest difference between the groups (coefficient = -2.40, q-value = 1.22x10^-2^). The significant MaAsLin2 results for all fixed effect covariates are available in **Supplementary Table S3**.

The combined results from machine learning, ANCOM-BC, and MaAsLin2, summarized in **Figure 5**, highlights areas of agreement and method-specific findings. *Cutibacterium acnes* SK182 was consistently identified as a control-associated taxon across all three methods, emphasizing its robustness as a key biomarker. For the cancer group, *Veillonella atypica* ACS-134-V-Col7a, *Corynebacterium ureicelerivorans* IMMIB RIV-2301, *Streptococcus suis* YS16, *Klebsiella pneumoniae* S_49BG, and *Prevotella melaninogenica* D18 were enriched for all three methods. While overlap was observed across methods, especially between the two differential abundance methods, certain taxa were uniquely identified by individual approaches. These unique features reflect differences in sensitivity and modeling assumptions for the three.

## 4 Discussion

The analysis conducted for this study revealed significant differences in the skin microbiome between cancer patients and individuals without cancer. Notably, the cancer groups exhibited increased alpha diversity compared to the control group. This observation aligns with broader research findings, where various studies have found elevated alpha diversity in non-skin microbiomes of cancer patients compared to controls (16, 17). Beta diversity analysis further supported these differences, with PERMANOVA results demonstrating statistically significant clustering between cancer and control groups.

In addition to the diversity measures, the machine learning model corroborated the differences in the study groups by achieving a mean F1 score of 0.943, which falls within the upper end of reported F1 scores (0.63 to 0.95) in recent microbiome sequencing studies (18–20). This performance highlights the model’s effectiveness in capturing relevant biological signals and suggests a significant distinction in the skin microbiomes of control and cancer subjects. Differential abundance analysis using ANCOM-BC and MaAsLin2 identified several taxa that were significantly enriched in either the cancer or control groups, supporting the machine learning findings and providing additional insights into the microbial shifts associated with cancer. The significant distinction observed between these groups supports the hypothesis that cancer is associated with dysbiosis in the skin microbiome. Dysbiosis, defined as an imbalance in the natural microflora of an individual, has been associated with a range of noncommunicable diseases in existing research. Further research could yield new therapeutic approaches for these diseases (21).

Of the three methods for identifying key taxa, including biomarker detection, ANCOM-BC, and MaAsLin2, six bacterial taxa were consistently identified across all methods. *Cutibacterium acnes* SK182 was the sole organism to be more strongly associated with the control group than the cancer group among these six key taxa. Furthermore, *Cutibacterium acnes* SK182 represented the highest magnitude of importance for all three analyses. The strain-level variations of *Cutibacterium acnes* (*C. acnes*) between the control and cancer group were particularly notable in the ML-derived biomarker detection results. *C. acnes* PMH5, a Type III *C. acnes* strain, was significantly associated with the cancer group. Type III strains are less commonly found on facial skin (22), such as the forehead and cheek swab sites used in this study. In contrast, Type IA strains, like *C. acnes* SK182, which are typically prevalent on the face (23), were strongly associated with the control group across all analyses. This shift in *C.acnes* phylotypes may suggest dysbiotic changes in the facial skin microbiome associated with cancer. However, the presence of another Type IA strain, *C. acnes* HL411PA1, in the cancer group indicates that these microbiome changes are complex and not uniform.

The five remaining key taxa, including Veillonella atypica, Corynebacterium ureicelerivorans, Streptococcus suis, Klebsiella pneumoniae, and Prevotella melaninogenica, were more strongly associated with the cancer group. Among these, Veillonella atypica has been previously linked to pancreatic cancer in studies of the gut microbiome, where it was enriched in pancreatic ductal adenocarcinoma (PDAC) patients compared to healthy controls (24). The identification of V. atypica in both gut and skin microbiomes suggests a potential systemic role for this taxon in pancreatic cancer. Similarly, Prevotella melaninogenica has been implicated in both oral cancer, where it has been proposed as a salivary biomarker for early detection (25), and in pancreatic cystic neoplasms, which may progress to pancreatic cancer through neoplastic transformation (26). These findings suggest that P. melaninogenica may have a broader role in cancer-associated dysbiosis across multiple cancer types, particularly in the early stages of disease progression.

Other identified key taxa may reflect opportunistic pathogenicity in immunocompromised cancer patients. In individuals undergoing chemotherapy, *Klebsiella pneumoniae* has been identified as a major complication, with mortality rates due to bacteremia ranging from 18% to 30% (27). Similarly, *Streptococcus suis*, although not commonly linked to human infections, has been associated with severe infections in immunosuppressed cancer patients (28). The final species, *Corynebacterium ureicelerivorans*, lacks specific links to cancer in the literature; however, at the genus level, *Corynebacterium* abundance has been observed to decrease in PDAC patients compared to healthy controls (29). The lack of specific findings at the species level demonstrates the need for further research.

While the data presents encouraging findings, this pilot study has several limitations. The sample size was small, and variations in the subject metadata may have introduced bias into the analysis, as suggested by the differences in beta diversity for age, gender, and number of medications. Additionally, some alpha diversity measures indicated higher diversity in samples from female subjects and those with higher chronological age, aligning with prior studies (30, 31). Further studies should account for these differences by incorporating a larger sample size with more uniformity in the subject demographics among the control and disease group.

The cheek and forehead microbiome profiling provided objective measurements; however, patient-reported information collected through the questionnaire, such as height and weight, may be subject to inaccuracy or variability, as this information was not supervised by medical personnel. While this patient-reported information was verified for completion upon submission, no additional steps were taken to assess its accuracy. Additionally, the status of skin health was documented in the patient questionnaire, but this data was not further analyzed. Skin health, in addition to current skin products and concrete medications, could have affected the microbiome at the time of collection. Current and previous therapeutic protocols, including chemotherapy, radiotherapy, and surgery, were also documented but not further analyzed. The time of sampling in relation to surgical and non-surgical therapy may have also affected the microbiome at the time of collection. Additionally, certain medications, such as antibiotics, greatly impact the microbiome and may have influenced the results reported in this study. Future larger studies should include additional analyses to investigate the relationship between skin health, other patient-reported factors, therapeutic interventions, medications, and the skin microbiome in both cancer and control cohorts.

This study relied on a single snapshot of the skin microbiome and lacked longitudinal data to observe changes over time with treatment or disease progression. Consistent staging of the condition may provide a clearer understanding of the skin microbiome during each stage of disease progression. Future research should prioritize multiple collections, including baseline or pre-treatment data, to better characterize skin microbiome shifts throughout treatment and disease progression. Additionally, further research is needed to investigate the underlying mechanisms that drive microbiome differences across various cancer stages, types, and microbiome sites. Such studies could provide opportunities to develop microbiome-based biomarkers that can identify pancreatic adenocarcinoma and other types of cancer.

In conclusion, analyzing the skin microbiome provides valuable insight into the diversity of organisms that are present in a person’s microflora and offers a framework to further examine the impact of dysbiosis in cancer.

## Data Availability

The raw data used in this article are not readily available, because of the proprietary nature of the primers, which are owned by ProdermIQ, Inc. Requests to access the raw data should be directed to the corresponding author (Erkut Borazanci, eborazanci@honorhealth.com).

## Supplementary material

Supplementary Table 1 ML-based biomarker detection results for selected taxa to predict cancer status.

Supplementary Table 2 ANCOM-BC results for taxa with significant differences between the control and cancer group.

Supplementary Table 3 MaAsLin2 results for taxa with significant differences by group, age, sample origin (no significant results), and gender covariates.
